# Nitric oxide-responsive interdomain regulation targets the σ^54^-interaction surface in the enhancer binding protein NorR

**DOI:** 10.1111/j.1365-2958.2010.07290.x

**Published:** 2010-09

**Authors:** Matthew Bush, Tamaswati Ghosh, Nicholas Tucker, Xiaodong Zhang, Ray Dixon

**Affiliations:** 1 Department of Molecular Microbiology, John Innes CentreNorwich Research Park, Colney NR4 7UH, UK; 2 Division of Molecular Bioscience, Imperial College LondonLondon SW7 2AZ, UK; 3 Strathclyde Institute of Pharmacy and Biomedical Sciences, University of Strathclyde161 Cathedral Street, Glasgow G4 0RE, UK

## Abstract

Bacterial enhancer binding proteins (bEBPs) are specialized transcriptional activators that assemble as hexameric rings in their active forms and utilize ATP hydrolysis to remodel the conformation of RNA polymerase containing the alternative sigma factor σ^54^. Transcriptional activation by the NorR bEBP is controlled by a regulatory GAF domain that represses the ATPase activity of the central AAA+ domain in the absence of nitric oxide. Here, we investigate the mechanism of interdomain repression in NorR by characterizing substitutions in the AAA+ domain that bypass repression by the regulatory domain. Most of these substitutions are located in the vicinity of the surface-exposed loops that engage σ^54^ during the ATP hydrolysis cycle or in the highly conserved GAFTGA motif that directly contacts σ^54^. Biochemical studies suggest that the bypass mutations in the GAFTGA loop do not influence the DNA binding properties of NorR or the assembly of higher order oligomers in the presence of enhancer DNA, and as expected these variants retain the ability to activate open complex formation *in vitro*. We identify a crucial arginine residue in the GAF domain that is essential for interdomain repression and demonstrate that hydrophobic substitutions at this position suppress the bypass phenotype of the GAFTGA substitutions. These observations suggest a novel mechanism for negative regulation in bEBPs in which the GAF domain targets the σ^54^-interaction surface to prevent access of the AAA+ domain to the sigma factor.

## Introduction

The promoter specificity of bacterial RNA polymerase is determined by the binding of an additional subunit, the sigma factor (σ). In contrast to the prototypical σ^70^ class of bacterial sigma factors, transcription by the σ^54^ class requires activation by bacterial enhancer binding proteins (bEBPs) that utilize nucleotide triphosphate hydrolysis to drive conformational rearrangements in the σ^54^-RNA polymerase holoenzyme. The central AAA+ domain of bEBPs is responsible for ATP hydrolysis and the consequent remodelling of the σ^54^-RNA polymerase that enables isomerization of the promoter DNA complexes from the closed to the open form (Wedel and Kustu, 1995, Cannon *et al*., 2000; Schumacher *et al*., 2004). As in the case of other AAA+ proteins, the σ^54^-interaction domain of bEBPs is competent for ATP hydrolysis when assembled as a hexameric ring (Rappas *et al*., 2007 and references therein). The bEBP subfamily of AAA+ domains contain specific structural features that enable nucleotide-dependent interactions with σ^54^. Most conserved amongst these is the GAFTGA motif, which forms a loop on the surface of the AAA+ domain that contacts σ^54^ during the ATP hydrolysis cycle (Bordes *et al*., 2003). Structural studies demonstrate that the GAFTGA loop (also known as the L1 loop), assisted by a second surface-exposed loop, L2, is in an extended conformation in the ATP bound transition state and is thus competent to engage with σ^54^. However, in the ADP bound state both loops are compacted towards the surface of the AAA+ domain enabling σ^54^ relocation, crucial to the conversion from the closed to the open complex (Rappas *et al*., 2006; Chen *et al*., 2007; Bose *et al*., 2008). The GAFTGA loop thus performs a crucial role in the ‘power stroke’ of bEBPs in coupling ATP hydrolysis to conformational rearrangements of the σ^54^-RNA polymerase.

Many bEBPs contain an amino-terminal regulatory domain that stringently controls the activity of the central AAA+ domain either negatively or positively in response to environmental cues (Studholme and Dixon, 2003). Most bEBPs also contain a helix–turn–helix DNA binding domain that binds to enhancer-like sequences upstream of promoters. In several well-characterized examples, allosteric control by the regulatory domain is exerted by controlling the oligomeric state of the AAA+ domain. In the ‘off’ state, the regulatory domain holds the AAA+ domain in an inactive dimeric form (Lee *et al*., 2003). Conformational changes in the regulatory domain induced by the signal enable transition to the ‘on’ state in which the AAA+ domain is released to form an active hexameric ring that is competent to activate transcription (Doucleff *et al*., 2005; De Carlo *et al*., 2006).

The nitric oxide (NO)-responsive bEBP, NorR, is required for transcriptional activation of the *norVW* genes in *Escherichia coli* that encode a flavorubredoxin and its associated NADH-dependent oxidoreductase respectively (Hutchings *et al*., 2002). These enzymes provide a detoxification system that reduces the NO radical to nitrous oxide under anaerobic conditions (Gardner *et al*., 2002; Gomes *et al*., 2002). Transcriptional activation by NorR is controlled by intramolecular interactions between an N-terminal regulatory GAF domain and the central AAA+ domain. The GAF domain contains a mononuclear non-haem iron centre that responds to NO through the formation of a mononitrosyl iron complex (D'Autreaux *et al*., 2005). In the absence of the NO signal, the GAF domain inhibits the activity of the AAA+ domain via interdomain repression (Gardner *et al*., 2003). Upon receipt of the signal and formation of the mononitrosyl iron species, repression of the AAA+ domain is relieved, activating ATP hydrolysis by NorR coupled to conformational remodelling of the σ^54^-RNA polymerase (D'Autreaux *et al*., 2005). In addition to allosteric control exerted by the GAF domain, our studies indicate that the C-terminal DNA binding domain of NorR plays a major role in the assembly of the functional AAA+ oligomer. Three enhancer sites located upstream of the *norVW* promoter are essential for transcriptional activation by NorR and provide a scaffold for the assembly of higher order oligomers (Tucker *et al*., 2010).

To investigate mechanisms of interdomain regulation in NorR, we have used a random mutagenesis approach to screen for mutations in the AAA+ domain that enable escape from GAF domain-mediated repression. Surprisingly, we find that substitutions within the highly conserved GAFTGA motif and in residues predicted to influence nucleotide-dependent conformational changes in this loop prevent intramolecular repression by the GAF domain in the absence of the NO signal. We demonstrate that the GAFTGA substitutions neither influence the DNA binding function of NorR nor the enhancer DNA-dependent oligomerization of the AAA+ domain and that variant proteins remain competent to catalyse open complex formation by σ^54^-RNA polymerase. Our results suggest that the σ^54^-interaction surface in the AAA+ domain is a target for intramolecular repression by the GAF domain.

## Results

### Mutations in the GAFTGA motif of NorR give rise to constitutive activity

**Fig. 1:**
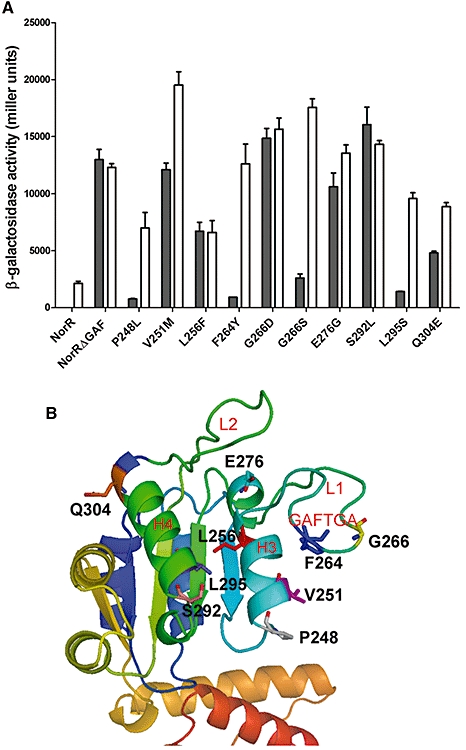
A. Transcriptional activation by NorR AAA+ domain variants *in vivo* as measured by the *norV–lacZ* reporter assay. Substitutions are indicated on the *x*-axis. ‘NorR’ refers to the wild-type protein and ‘NorRΔGAF’ refers to the truncated form lacking the GAF domain (residues 1–170). Cultures were grown either in the absence (black bars) or presence (white bars) of 4 mM potassium nitrite, which induces endogenous NO production. Error bars show the standard error of the three replicates carried out for each condition. B. Structural model of the AAA+ domain of NorR based on the NtrC1 structure (Lee *et al*., 2003) (1NY5 chain A). The helices and loops (H3 and H4, L1 and L2) involved in nucleotide-dependent conformational changes in bEBPs are labelled in red. Residues that were substituted as a consequence of the PCR mutagenesis of the AAA+ domain are indicated. The F264 and G266 residues form part of the GAFTGA motif that contacts σ^54^.

To explore the mechanism of interdomain repression in NorR, error-prone polymerase chain reaction (PCR) mutagenesis was employed to create mutations that potentially disrupt repression of the AAA+ domain by the N-terminal (NO-sensing) GAF domain. This strategy produced mutant versions of NorR that had significant activity in cultures grown in the absence of an NO source, in contrast to wild-type NorR, which is activated by endogenous NO generated in the presence of potassium nitrate (Fig. 1A). In some cases (e.g. G266D, S292L) activity in the absence of the signal was similar to that exhibited by a truncated version of NorR lacking the GAF domain (NorRΔGAF). This phenotype suggests that repression by the GAF domain has been bypassed, resulting in loss of regulation upon the AAA+ domain. In other cases (e.g. F264Y, Q304E) some repression in the absence of NO was evident, indicative of a partial bypass phenotype. In structural models of the AAA+ domain of NorR based on the structure of NtrC1 (Lee *et al*., 2003), the majority of the substitutions are located in either helix 3 (H3), helix 4 (H4) or loop 1 (L1) (Fig. 1B). These are the structural features in the AAA+ domain that undergo nucleotide-dependent conformational changes during the ATPase cycle to promote engagement with σ^54^. For example, the equivalent of E276 in PspF (E97) is located close to the base of the L1 loop and forms nucleotide-dependent interactions with R131 (equivalent to NorR R310) in the L2 loop that co-ordinate loop movements during ATP hydrolysis (Rappas *et al*., 2006). The only substitution predicted to be located outside this region of nucleotide-induced conformational change is Q304; where the equivalent residue in NtrC1 is most probably involved in inter AAA+ domain subunit interactions. Significantly, three substitutions were identified within the highly conserved GAFTGA motif itself. The most notable of these was the G266D mutation, located in the second glycine of the motif, which allowed full escape from the GAF-mediated repression of NorR activity (Fig. 1A). This is surprising given that this loop is required to contact σ^54^ to drive open complex formation (Buck *et al*., 2006) and that substitutions at G266 are likely to influence the conformational flexibility of this loop. In order to examine which amino acids at the G266 position give rise to constitutive activity, we substituted this residue for each of the other 19 natural amino acids (Fig. S1). In addition to the aspartate substitution that gives rise to constitutive activity; asparagine, glutamine, serine, cysteine and methionine all gave activity in the absence of an NO source. Asparagine and aspartate changes gave fully constitutive phenotypes whereas the other changes were still partially subject to regulation by the GAF domain. Surprisingly, the glutamate substitution did not produce a functional NorR protein. The remaining amino acid changes all resulted in non-functional proteins and Western blotting confirmed that this is not due to instability (data not shown). The non-functional nature of most substitutions at this position is not unexpected, given the importance of the GAFTGA motif and its high conservation in bEBPs.

The apparent loss of regulation in the G266D and G266N variants suggest that these mutations completely bypass the repressive function of the N-terminal GAF domain. To confirm that the NO-sensing function of the GAF domain no longer contributes to the phenotype of the G266D variant, targeted substitutions were made at residues known to disrupt the non-haem iron centre in the GAF domain (Tucker *et al*., 2008). When the R75K, Y98L, C113S, H111Y or D99A substitutions were combined with G266D, no reduction in the ability to activate transcription by NorR was observed (Fig. S2). To further test the influence of the GAF domain in this variant, the sequence encoding the first 170 residues of NorR was deleted in constructs containing an additional N-terminal, hexa-histidine tag. The resulting G266DΔGAF–His protein was comparable with the G266D–His variant in its ability to activate transcription *in vivo* (Fig. S3). This was also true for the G266N–His protein. The Q304E variant in contrast showed a partial bypass phenotype (Fig. 1A) and removal of the GAF domain led to constitutive activity as anticipated (Fig. S3).

### The G266D mutation does not affect enhancer binding or oligomerization of NorR *in vitro*

**Fig. 2:**
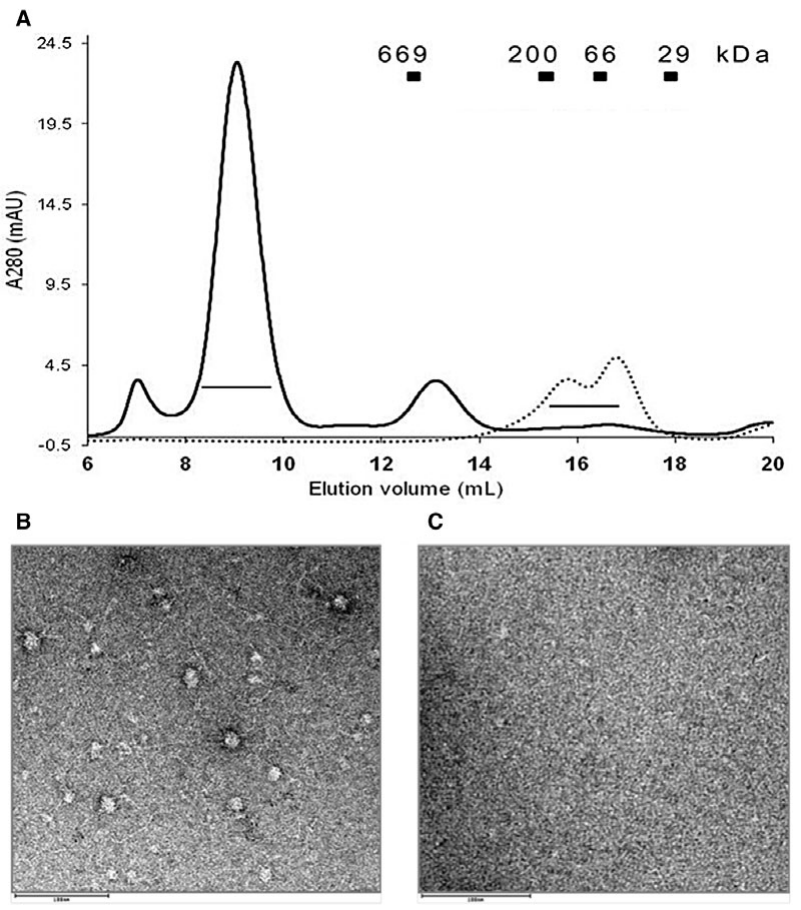
Enhancer-dependent higher order oligomeric assembly of the G266DΔGAF-His variant. A. Gel filtration chromatography of 9 µM G266DΔGAF–His variant in the absence (*dotted line*) and presence (*solid line*) of 0.75 µM 266 bp dsDNA (molar ratio of 12:1 monomer: DNA), containing all three enhancer sites, performed at 4°C using a Superose 6 column (24 ml). The presence of DNA stabilizes a higher order oligomeric form of G266DΔGAF–His. The lines below the elution peaks represent the fractions analysed by negative-stain electron microscopy. Corresponding molecular weight of standard globular proteins are indicated at their elution volume. B and C. Negative-stain electron microscopy studies. Shown are raw micrographs of G266DΔGAF-His alone (C) and in complex with 266 bp DNA (B), scale bar 100 nm. Ring-shaped oligomeric particles were only observed in the presence of DNA.

Since the oligomerization state and hence the activity of the AAA+ domain of bEBPs is often controlled by regulatory domains, we questioned whether the NorR GAFTGA substitutions might bypass the repressive function of the GAF domain by altering the assembly of higher order oligomers. Since binding of NorR to enhancer sites is essential for the formation of stable oligomers and enhancer DNA appears to be a key ligand in the activation of NorR as a transcription factor (Tucker *et al*., 2010), we first investigated whether the GAFTGA mutations influence DNA binding. For this and subsequent biochemical experiments we used GAF domain deleted forms of NorR (NorRΔGAF) and utilized N-terminal histidine tags as an aid to protein purification. The presence of this tag does not significantly affect the activity of wild-type NorRΔGAF or its variants *in vivo* (data not shown). We observed that the affinity of NorRΔGAF for a 361 bp DNA fragment containing the three enhancer sites upstream of the *norV* promoter was not significantly influenced by the presence of the G266D and G266N substitutions (Fig. S4). Dissociation constants (Kd) were calculated as 100 nM in each case. To determine the effect of the G266D substitution on enhancer-dependent NorR oligomer formation (Tucker *et al*., 2010), we performed analytical gel filtration experiments in the absence and presence of a 266 bp DNA fragment containing the three enhancer sites. Based on reference elution volumes obtained with different protein standards, unbound G266DΔGAF–His eluted as an apparent monomer/dimer species (Fig. 2A). The presence of the 266 bp DNA fragment shifted the protein peak towards a higher molecular mass species (Fig. 2A) indicating formation and stabilization of a higher order nucleoprotein complex. These elution profiles are similar to that reported recently for wild-type NorRΔGAF (Tucker *et al*., 2010). Analysis of the purified protein–DNA complex using negatively stained electron microscopy, allowed visualization of higher order ring-shaped particles with dimensions of 125 Å in diameter (Fig. 2B) consistent with a hexameric ring observed for NorRΔGAF in complex with the 266 bp DNA fragment (Tucker *et al*., 2010). No oligomeric particles were seen in the electron micrographs for protein alone (Fig. 2C). We conclude from these studies that the G266D mutation does not apparently influence the oligomeric assembly of the AAA+ domain or the requirement for enhancer sites to stabilize the formation of a higher order oligomer.

### G266 bypass variants show enhancer-dependent ATPase activity *in vitro*

**Fig. 3:**
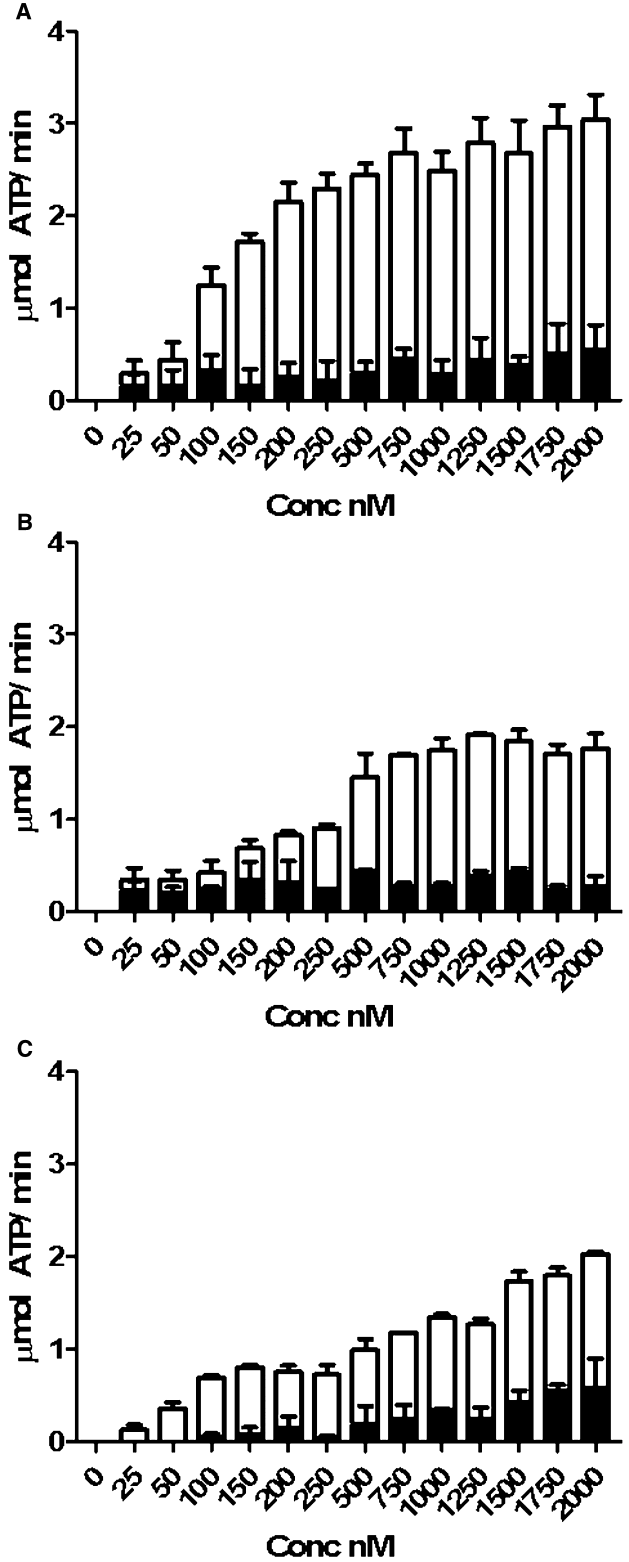
ATPase activity of the NorRΔGAF–His (A), G266DΔGAF–His (B) and G266NΔGAF–His (C) variants in response to protein concentration and the presence of enhancer DNA. Assays were conducted either in the absence (closed bars) or presence (open bars) of the 266 bp DNA fragment (final concentration 5 nM) that includes the *norR–norVW* intergenic region and each of the three NorR binding sites. Data are shown as the mean from at least two experiments.

In bEBPs the ATP hydrolysis site is configured through interactions between adjacent AAA+ protomers in the hexameric ring (Schumacher *et al*., 2008). Since the GAFTGA motif relays nucleotide-dependent interactions at this site to enable contact with σ^54^, we were interested to examine if the G266 substitutions influence ATPase activity. We have already established that enhancer DNA is required for ATP hydrolysis by NorR and that the three binding sites upstream of the *norV* promoter are necessary for activation of ATPase activity, consistent with the requirement for DNA for formation of a functional higher order oligomer (Tucker *et al*., 2010). Using concentrations of NorRΔGAF–His within the anticipated physiological range, we observed low levels of ATP hydrolysis in the absence of enhancer DNA. This was also a property of the G266D and G266N variants (Fig. 3B and C, black bars). Consistent with our previous studies with a non-his-tagged form of NorRΔGAF, ATPase activity was strongly stimulated by the presence of promoter DNA. Under these conditions ATP hydrolysis by NorRΔGAF–His increased as a sigmoidal reponse to increasing protein concentration indicative of positive cooperativity, with a lower rate of increase exhibited at concentrations above 250 nM (Fig. 3A, white bars). The absence of increased activity at higher protein concentrations may reflect saturation of the enhancer sites consistent with the observed DNA binding constant (100 nM as reported above, Fig. S4). Although ATP hydrolysis by the G266D and G266N variants was also stimulated by the enhancer sites, the response to protein concentration was less cooperative than observed with NorRΔGAF–His and activities were lower than those of the wild-type protein even at a relatively high protein concentration (2 uM). Since the enhancer DNA is likely to be fully saturated with protein at concentrations above 300 nM, the G266 substitutions may alter the stability of the nucleoprotein complexes, perhaps by influencing protomer interactions that impact upon the ATP hydrolysis site.

### The GAFTGA variants can activate open complex formation *in vitro*

**Fig. 4:**
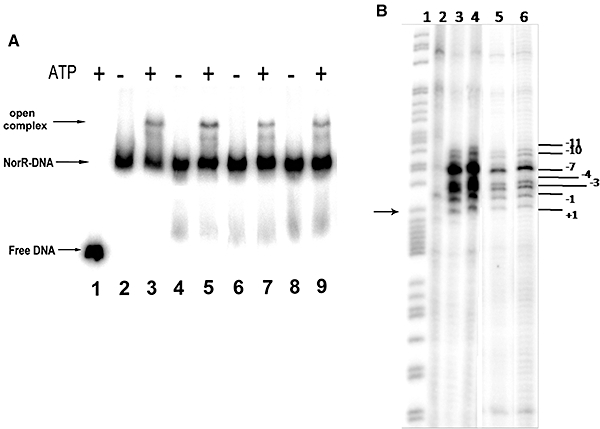
Open promoter complex formation by AAA+ variants. A. Heparin-resistant complexes formed by NorRΔGAF, NorRΔGAF–His, G266DΔGAF–His and G266NΔGAF–His, on the 361 bp DNA fragment carrying the *norR–norVW* intergenic region. In all cases, the final NorR concentration was 1500 nM. Reactions contained no NorR (lane 1), NorRΔGAF (lanes 2 and 3), NorRΔGAF–His (lanes 4 and 5), G266DΔGAF–His (lanes 6 and 7) and G266NΔGAF–His (lanes 8 and 9). Reactions loaded in lanes 1, 3, 5, 7 and 9 contained ATP (final concentration 5 mM), which was absent in lanes 2, 4, 6 and 8. Arrows indicate the position of free DNA, NorR bound DNA and the open promoter complexes. B. Potassium permanganate footprinting of the 266 bp *norR–norVW* promoter fragment after open complex formation initiated by NorR. Lane 1 is a G+A ladder. Lane 2 is a control without activator present. Lanes 3, 4, 5 and 6 show footprinting after initiation of open complexes in the presence of 1 µM (final concentration) ΔGAF, ΔGAF–His, G266DΔGAF–His and G266NΔGAF–His respectively. The arrow marks the *norVW* transcriptional start and the positions of the enhanced cleavage at T bases are indicated.

To further test the functionality of the G266 variants *in vitro*, we conducted assays to measure their ability to catalyse the conversion of the σ^54^-RNA polymerase closed complexes to open promoter complexes. Although NorR–DNA complexes exhibit heparin resistance, open promoter complexes can be visualized as heparin-resistant super-shifted species on non-denaturing gels (D'Autreaux *et al*., 2005). In the presence of all the components required for open complex formation, the G266D and G266N variants were competent to form the super-shifted species, as in the case of NorRΔGAF (Fig. 4A compare lanes 3, 5, 7 and 9). Open complex formation was ATP-dependent as expected (Fig. 4A lanes 2, 4, 6 and 8). In order to probe the nature of the open complexes formed, we footprinted complexes with potassium permanganate, which targets cleavage to single stranded DNA regions, hence providing sequence-specific information. In all cases, we observed enhanced cleavage corresponding to T residues located between −11 and +1 in the *norV* promoter, consistent with the expected footprint. Notably, the band intensity observed with the G266 variants was decreased in comparison with NorRΔGAF or NorRΔGAF–His, perhaps reflecting the lower ATPase activities exhibited by the GAFTGA variants when compared with the wild type. These results confirm that the G266 variants are competent to interact with σ^54^ and can activate transcription *in vitro*, even though they exhibit altered ATPase activities.

### Evidence for direct intramolecular interaction between the GAF domain and the σ^54^-interaction surface

From the biochemical results presented thus far, it seems likely that the GAFTGA mutations do not bypass intramolecular repression solely on the basis of changes in oligomerization state. To gain more insight into the nature of the interactions between the GAF and AAA+ domains, we followed a genetic suppression strategy. In previous work, mutagenesis of conserved residues in the GAF domain identified the R81L change that allows partial escape from interdomain repression in NorR (Tucker *et al*., 2008). To further investigate the role of this residue in the regulation of AAA+ activity, a number of other changes were made at this position (Fig. S5). *In vivo* assays for transcriptional activation by NorR showed that the R81 residue is critical for the negative regulation of the AAA+ domain by the GAF domain. Hydrophobic changes (including R81L) result in significant constitutive activity. Negatively charged residues and serine substitutions not only prevent negative control but also stimulate NorR activity beyond wild-type levels. R81D, R81N and R81E give rise to twofold to threefold more activity than NorRΔGAF.

**Fig. 5:**
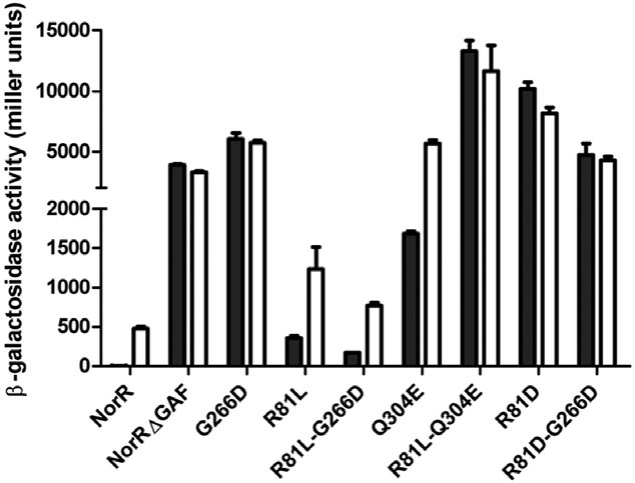
Suppression of the G266D variant phenotype by the R81L mutation as measured by the *norV–lacZ* reporter assay *in vivo*. Substitutions are indicated on the *x*-axis. ‘NorR’ refers to the wild-type protein and ‘NorRΔGAF’ refers to the truncated form lacking the GAF domain (Δ1–170). Cultures were grown either in the absence (black bars) or presence (white bars) of 4 mM potassium nitrite, which induces endogenous NO production. Error bars show the standard error of the three replicates carried out for each condition.

Since the R81 residue appears to be critical for interdomain repression, we decided to investigate whether R81 is required for positioning the GAF domain in the vicinity of the GAFTGA motif. We observed that the R81L substitution suppresses the constitutive activity of the G266D mutant so that repression of the AAA+ domain is almost completely restored (Fig. 5). Interestingly, the R81L mutation has a similar effect on other constitutively active variants located in the key region of the AAA+ domain that is predicted to undergo conformational changes upon ATP hydrolysis (Fig. S6A). As mentioned above, the Q304 residue is predicted to be at the base of helix 4 in the AAA+ domain of NorR and is not expected to have a role in co-ordinating movements in the GAFTGA loop upon transition to the ‘on’ state. In accordance with this, the Q304E mutation was not suppressed by the R81L substitution. Instead, when combined with Q304E, the R81L substitution enabled complete escape from interdomain repression (Fig. 5).

Next, we wanted to determine whether the suppression of the G266D phenotype was dependent on the substitution made at the R81 position. Results show that only hydrophobic changes including R81L, V, I and F enable suppression (Fig. S7). It is possible that such changes introduce a new hydrophobic contact that helps restore interactions between the GAF and AAA+ domains. Western blotting analysis shows that reduction in activity is not due to a decrease in the stability of these double mutants (data not shown). Moreover other substitutions such as R81D have no effect on the constitutive activity of the G266D NorR variant (Fig. 5). Overall, the constitutive activity of the G266D variant and the specific suppression of this phenotype by hydrophobic changes at the R81 position suggest that the GAF domain may target the GAFTGA motif to prevent σ^54^ contact in the absence of the NO signal. Furthermore, the R81 residue is critical in maintaining repression and may be a key residue in mediating the transition from the ‘off’ to the ‘on’ state.

## Discussion

**Fig. 6:**
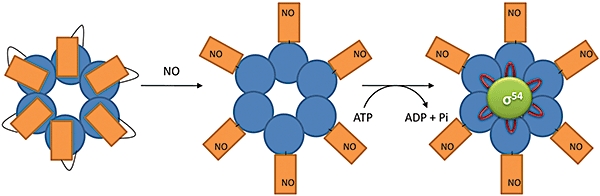
Model for regulation of σ^54^-dependent transcription by the EBP NorR. Binding of NorR to the *norR–norVW* intergenic region that contains the three NorR binding sites (not shown) is thought to facilitate the formation of a higher order oligomer that is most likely to be a hexamer (Tucker *et al*., 2010). In the ‘off’ state, the N-terminal GAF domains (orange rectangles) negatively regulate the activity of the AAA+ domains (blue circles) by preventing access of the L1 and L2 loops to σ^54^ (left). In the ‘on’ state, NO binds to the iron centre in the GAF domain forming a mononitrosyl iron species. The repression of the AAA+ domain is relieved (centre), enabling ATP hydrolysis by NorR coupled to conformational changes in the AAA+ domain. During the nucleotide hydrolysis cycle, the surface-exposed loop that includes the GAFTGA motif moves into an extended conformation to allow σ^54^-interaction and remodelling (right).

The lack of NO-responsive regulation in truncated forms of NorR that lack the GAF domain (D'Autreaux *et al*., 2005), clearly places NorR in the class of bEBPs that are negatively regulated. The substitutions we have identified in the AAA+ domain that bypass negative control by the GAF domain, cluster in regions that modulate the conformation of the σ^54^-interaction surface or in the conserved GAFTGA motif itself. This invokes a model whereby the GAF domain negatively regulates the AAA+ domain by preventing access of the L1 and L2 loops to σ^54^ (Fig. 6). This mode of repression might also serve to lock the loops in a restrained conformation that feeds back to the nucleotide binding site to prevent ATP hydrolysis. According to this model, substitutions in the σ^54^-interaction surface bypass negative regulation either by altering the conformation of this surface to restrict access by the GAF domain or by directly disrupting GAF–AAA+ domain interactions. The alternative explanation that these substitutions bypass negative control by locking the AAA+ domain in a constitutive hexameric oligomerization state seems unlikely given that the GAFTGA substitutions exhibited no major changes in oligomerization properties when examined in the context of the NorRΔGAF protein. Although the full-length NorR apoprotein is competent to bind enhancer DNA this nucleoprotein complex is inactive with respect to ATP hydrolysis and transcriptional activation (D'Autreaux *et al*., 2005). This suggests that in the absence of the NO signal, the GAF domain maintains the nucleoprotein complex in an inactive state by preventing access to σ^54^-RNA polymerase.

It is remarkable that substitutions in the surface-exposed GAFTGA loop are able to prevent negative regulation by the GAF domain but still retain the ability to interact with σ^54^ and activate open complex formation. In the majority of bEBPs, substitutions in the GAFTGA motif cause a severe defect on the ability of the protein to activate transcription (Zhang *et al*., 2002 and references cited therein). In PspF, the conserved threonine in this loop plays a critical role in contacting σ^54^ (Bordes *et al*., 2003; Dago *et al*., 2007) and only substitution of tyrosine for the highly conserved phenylalanine permits transcriptional activation (Zhang *et al*., 2009). We observe that the equivalent aromatic substitution in NorR, F264Y, allows partial escape from repression by the GAF domain. Few studies have been carried out to explore the role of the second glycine in the GAFTGA motif. In NtrC, the G219K variant has improved DNA binding activity and ATPase activity is 50% of the activated wild type (North *et al*., 1996). However, it fails to activate transcription (a property exhibited by the equivalent mutation in NorR, G266K), suggesting that this mutation may prevent the interaction with σ^54^. In contrast, the G219C variant of NtrC is competent to form open complexes but intriguingly can only do so in the absence of enhancer DNA (Yan and Kustu, 1999). This defect may be explained by changes in the relative juxtaposition of the DNA binding and ATPase domains observed during the ATPase cycle (De Carlo *et al*., 2006). Overall, positively charged or aromatic residues are apparently not tolerated at this position in NorR, which may reflect a requirement for the σ^54^-interaction.

The role of the σ^54^-interaction surface in negative regulation by the GAF domain is further supported by our suppression data. The R81 residue in the GAF domain apparently plays a critical role in the mechanism of interdomain repression since an alanine substitution at this position leads to constitutive activation, whereas hydrophobic substitutions, particularly leucine, restore repression only when combined with specific bypass mutations in the AAA+ domain, including those in the GAFTGA loop. Structural modelling of the GAF domain, suggests that the R81 residue is surface-exposed (Tucker *et al*., 2008). It is located at the opposite end of an α-helix to the R75 residue (Fig. S8), which is postulated to be a ligand to the hexa-coordinated iron and is the most suitable candidate to be displaced upon NO binding (Tucker *et al*., 2008). Therefore, it is possible that formation of the mononitrosyl iron complex would displace the R75 ligand causing a conformational change in the helix that repositions R81. Interactions between the R81 residue and residue(s) in the AAA+ domain may thus facilitate the switch from the ‘off’ to the ‘on’ state.

The results presented here suggest a novel mechanism for negatively regulating bEBPs in which the σ^54^-interaction surface is a target for repression, rather than the assembly of the active higher order oligomer. In the response regulator bEBPs NtrC1 and DctD, interactions between the receiver domain and the AAA+ domain maintain these proteins as inactive dimers in the absence of a regulatory signal. Extensive interdomain contacts, established via a coiled-coil linker, hold the ATPase domains in a dimeric front-to-front configuration to prevent oligomerization. Upon phosphorylation the dimerization interface in the receiver domain, which includes the coiled-coil linker is disrupted, allowing the AAA+ domain to reorient into the front-to-back configuration required for assembly into the active oligomeric ring (Lee *et al*., 2003; Doucleff *et al*., 2005). The linker region between the GAF and AAA+ domains of NorR is not predicted to form a coiled-coil helix, a structural feature that is also absent in negatively regulated NtrC4 and positively regulated NtrC (Batchelor *et al*., 2008). NtrC4 has a partially disrupted receiver–AAA+ domain interface and can assemble into active oligomers at high protein concentrations independent of phosphorylation, a process that does not occur with NtrC1 (Batchelor *et al*., 2008). The activated receiver domain has been shown to stabilize the hexameric form of NtrC4, thus functioning as an intermediate between the negative mechanism of NtrC1/DctD and positive mechanism of NtrC (Batchelor *et al*., 2008; Batchelor *et al*., 2009). In some bEBPs, the activity of the AAA+ domain is controlled by another regulatory protein, rather than by intramolecular repression (e.g. NifA, PspF, HrpR/S). In the case of PspF, which does not contain an amino-terminal regulatory domain, the activity of the AAA+ domain is negatively controlled by the PspA protein. In this case, repression is neither achieved by controlling the assembly of the ATPase subunits nor by preventing access of PspF to σ^54^, but rather by inhibition of ATP hydrolysis (Joly *et al*., 2009). Inhibition is mediated by the interaction of PspA with a surface-exposed tryptophan residue (W56) on PspF, which is likely to communicate with the ATP hydrolysis site. Structural studies have identified N64 in the AAA+ domain of PspF as being the key residue that translates nucleotide hydrolysis to conformational changes (Rappas *et al*., 2006) and links ligand binding to ATPase activity (Zhang and Wigley, 2008). Although N64 variants are still able to bind PspA, their ATPase activity is no longer inhibited (Joly *et al*., 2008) suggesting that negative regulation by PspA at the W56 residue is directly signalled to the nucleotide machinery via N64 to prevent ATPase hydrolysis by PspF. NorR represents another mechanism of negative regulation in which the N-terminal regulatory domain targets the σ^54^-interacting region of the AAA+ domain that includes the GAFTGA motif. The evolutionary and physiological advantages of these different modes of regulation in bEBPs remain to be elucidated. In the case of NorR, we speculate that pre-assembly of an inactive oligomeric NorR species, poised as a nucleoprotein complex at the enhancer sites, enables the cell to rapidly respond to NO stress.

## Experimental procedures

### Plasmids and site directed mutagenesis

The pMJB1 plasmid was constructed from the pNorR plasmid (Tucker *et al*., 2004) by making two silent mutations within the *norR* sequence. The C496T mutation produced the *Mfe*I/*Mun*I restriction site (CAATTG) upstream of the AAA+ domain and the G1341C mutation produced the *Sst*II restriction site (CCGCGG) downstream. In all other cases, targeted mutagenesis of the *norR* sequence was carried out using a PCR method (Ito *et al*., 1991) with pMJB1 as a template.

### Random mutagenesis

Random PCR mutagenesis was carried out with *Taq* DNA polymerase under standard reaction conditions. Reaction mixtures contained 75 ng of template pMJB1, 100 ng of each primer (AAA+Fwd 5′-GAAGAGCTACGGCTGATTGC-3′ and AAA+Rev 5′-GAACGCTTCTGTCGCTTCAC-3′), 0.2 mM dNTPs, 1.5 mM MgCl_2_ and 5 units of enzyme in a final volume of 50 ml. The PCR products were purified, digested with *Mfe*I and *Sst*II and subsequently recloned into pMJB1 digested with the same enzymes. Ethanol precipitation followed by electroporation of the ligated mutant plasmid sample into DH5α was conducted and plasmid purification carried out before transformation of the sample into MH1003 (Hutchings *et al*., 2002). Transformants were screened on Luria–Bertani (LB) supplemented with Xgal (40 µg ml^−1^), chloramphenicol (30 µg ml^−1^), carbenicillin (100 µg ml^−1^) and kanamycin (50 µg ml^−1^). Constitutive mutants were identified based on the ability of the *norR* gene to produce a protein that can activate expression of a *norV–lacZ* fusion. In the absence of inducer, constitutive mutants activate expression of β-galactosidase that cleaves the Xgal substrate to produce a blue product.

### Assaying NorR activity *in vivo*

Transcriptional activation by NorR *in vivo* was measured by introducing wild-type and mutant plasmids into MH1003 a *nor*: : *cat* derivative of *E. coli* strain MC1000 with a *lacZ* reporter fusion to the *norVW* promoter inserted at the phage λ attachment site (Hutchings *et al*., 2002). Cultures were grown with shaking in 50 ml of LB medium at 37°C until the OD650 reached 0.3, at which point glucose was added to the culture to a final concentration of 1%. Cultures were then split into 8 ml Bijou bottles and were grown anaerobically overnight at 37°C with or without potassium nitrite (4 mM). Under the latter conditions, NorR is activated by the NO that is generated endogenously by nitrite reduction in *E. coli* (Hutchings *et al*., 2002). Levels of expression of the *norV–lacZ* fusion were then determined by assaying β-galactosidase activity as previously described (Tucker *et al*., 2008).

### Protein purification

*Escherichia coli* K12 NorRΔGAF was overexpressed and purified as described previously (D'Autreaux *et al*., 2005). NorRΔGAF and mutant derivatives were additionally purified via an N-terminal TEV cleavable His-tag. Proteins were overexpressed from the pET-M11 construct but with the NcoI site altered to an NdeI site, to allow easy cloning of the *norR* sequence. BL21(DE3) transformed with the relevant construct was grown shaking at 250 r.p.m. at 30°C to an OD600 of 0.6. IPTG was then added to a final concentration of 1 mM and the cells left for 2–3 h before harvesting at 5000 r.p.m. Pellets were resuspended in buffer A (100 mM Tris-Cl, 50 mM NaCl, 50 mM imidazole, 5% glycerol, pH 8.5) containing EDTA-free protease inhibitors (Roche) and the cells were broken by French pressure disruption (1000 psi) in two passes. The insoluble material was then removed by centrifugation at 15 000 r.p.m. for 30 min. The clarified supernatant was loaded onto two 1 ml HiTrap chelating HP columns, connected in series and charged with 100 mM nickel chloride. The columns were equilibrated with NorR buffer A. Protein was then eluted using NorR buffer B (100 mM Tris-Cl, 50 mM NaCl, 500 mM imidazole, 5% glycerol, pH 8.5). To remove imidazole and to prevent precipitation, NorR containing fractions were loaded as quickly as possible onto a Superdex 200 16/60 column (Amersham Biosciences), pre-equlibrated in buffer C (100 mM Tris-Cl, 200 mM Nacl, 8 mM DTT, 5% glycerol). NorR containing fractions were concentrated using Amicon Ultra (Millipore) centrifugal devices with a 30 kDa MWC, aliquoted and stored in buffer containing 100 mM Tris-Cl, 100 mM NaCl, 4 mM DTT and 40% glycerol at −80°C, until required.

### Open promoter complex and gel retardation assays

Open complex and gel retardation assays were carried out as described previously (Tucker *et al*., 2010) using fragments derived from the pNPTprom plasmid that contains the *norR–norVW* region blunt-end cloned into the SmaI site of pUC19 (Tucker *et al*., 2004).

### Potassium permanganate footprinting of open complexes

Open complexes were probed using potassium permanganate as described previously (Whitehall *et al*., 1992). Following potassium permanganate treatment, samples were resuspended in sodium acetate before ethanol precipitation. Samples were then subjected to chemical cleavage using the Maxam and Gilbert method. A G+A sequencing ladder was prepared by treatment with formic acid prior to the same cleavage treatment. The footprinting fragments were dried and dissolved in sequencing dye before being loaded on a sequencing gel.

### ATPase assays

ATPase activities were measured using an assay in which production of ADP is coupled to the oxidation of NADH by lactate dehydrogenase and pyruvate kinase (Norby, 1988). The oxidation of NADH was monitored at 340 nm at 37°C. All reaction mixtures contained ATP (30 mM), phosphoenolpyruvate (1 mM), NADH (0.3 mM), pyruvate kinase (7 U, Roche), lactate dehydrogenase (23 U, Roche) in 50 mM Tris-HCl (pH 8.0), 100 mM KCl, 2 mM MgCl. Increasing volumes of NorR–His and its variants were added and the ATPase activity was measured by observing the change in absorbance at 340 nm. Total activity (µmol ATP min^−1^) at each concentration was calculated using the equation: [(ΔOD340/Δt)/6220]*1 × 10^6^ where t is the time-course of the experiment in minutes. Reactions were carried out both in the absence and presence of 5 nM of a 266 bp fragment of the *norR–norVW* intergenic region generated from the pNPTprom plasmid (Tucker *et al*., 2004) using the norRpromF (5′-GGCGATATTCGCCAGCACAT-3′) and norRpromR (5′-CGTTGACCAACCCAATGAATGT-3′) primers.

### Analytical gel filtration

Gel filtration chromatography of G266DΔGAF–His protein alone and in complex with a 266 bp DNA fragment, containing all three enhancer sites, was performed using a Superose 6 column (10 × 300 mm, 24 ml) as described previously (Tucker *et al*., 2010). The DNA fragment was generated by PCR as described previously (Tucker *et al*., 2010).

### Negative-stain electron microscopy

Samples (2 µl) from fractions eluted from gel filtration columns containing either G266DΔGAF–His alone or in complex with 266 bp dsDNA were absorbed onto glow-discharged continuous carbon grids (TAAB) and stained with 2% uranyl acetate. Data were collected at 35 000× magnification using a FEI Tecnai 12 electron microscope operating at 120 kV. Micrographs were recorded directly on a 1 k × 1 k CCD camera (TVIPS, Germany).

## References

[b1] Batchelor JD, Doucleff M, Lee C-J, Matsubara K, De Carlo S, Heideker J (2008). Structure and regulatory mechanism of aquifex aeolicus NtrC4: variability and evolution in bacterial transcriptional regulation. J Mol Biol.

[b2] Batchelor JD, Sterling HJ, Hong E, Williams ER, Wemmer DE (2009). Receiver domains control the active-state stoichiometry of aquifex aeolicus [sigma]54 activator NtrC4, as revealed by electrospray ionization mass spectrometry. J Mol Biol.

[b3] Bordes P, Wigneshweraraj SR, Schumacher J, Zhang X, Chaney M, Buck M (2003). The ATP hydrolyzing transcription activator phage shock protein F of *Escherichia coli*: identifying a surface that binds sigma 54. Proc Natl Acad Sci USA.

[b4] Bose D, Pape T, Burrows PC, Rappas M, Wigneshweraraj SR, Buck M, Zhang X (2008). Organization of an activator-bound RNA polymerase holoenzyme. Mol Cell.

[b5] Buck M, Bose D, Burrows P, Cannon W, Joly N, Pape T (2006). A second paradigm for gene activation in bacteria. Biochem Soc Trans.

[b6] Cannon WV, Gallegos MT, Buck M (2000). Isomerization of a binary sigma-promoter DNA complex by transcription activators. Nat Struct Biol.

[b7] Chen B, Doucleff M, Wemmer DE, De Carlo S, Huang HH, Nogales E (2007). ATP ground- and transition states of bacterial enhancer binding AAA+ ATPases support complex formation with their target protein, σ^54^. Structure.

[b8] D'Autreaux B, Tucker NP, Dixon R, Spiro S (2005). A non-haem iron centre in the transcription factor NorR senses nitric oxide. Nature.

[b9] Dago AE, Wigneshweraraj SR, Buck M, Morett E (2007). A role for the conserved GAFTGA motif of AAA+ transcription activators in sensing promoter DNA conformation. J Biol Chem.

[b10] De Carlo S, Chen B, Hoover TR, Kondrashkina E, Nogales E, Nixon BT (2006). The structural basis for regulated assembly and function of the transcriptional activator NtrC. Genes Dev.

[b11] Doucleff M, Chen B, Maris AE, Wemmer DE, Kondrashkina E, Nixon BT (2005). Negative regulation of AAA+ ATPase assembly by two component receiver domains: a transcription activation mechanism that is conserved in mesophilic and extremely hyperthermophilic bacteria. J Mol Biol.

[b12] Gardner AM, Helmick RA, Gardner PR (2002). Flavorubredoxin, an inducible catalyst for nitric oxide reduction and detoxification in *Escherichia coli*. J Biol Chem.

[b13] Gardner AM, Gessner CR, Gardner PR (2003). Regulation of the nitric oxide reduction operon (*norRVW*) in *Escherichia coli*. Role of NorR and sigma54 in the nitric oxide stress response. J Biol Chem.

[b14] Gomes CM, Giuffre A, Forte E, Vicente JB, Saraiva LM, Brunori M, Teixeira M (2002). A novel type of nitric-oxide reductase. *Escherichia coli* flavorubredoxin. J Biol Chem.

[b15] Hutchings MI, Mandhana N, Spiro S (2002). The NorR protein of *Escherichia coli* activates expression of the flavorubredoxin gene norV in response to reactive nitrogen species. J Bacteriol.

[b16] Ito W, Ishiguro H, Kurosawa Y (1991). A general method for introducing a series of mutations into cloned DNA using the polymerase chain reaction. Gene.

[b17] Joly N, Burrows PC, Buck M (2008). An intramolecular route for coupling ATPase Activity in AAA+ proteins for transcription activation. J Biol Chem.

[b18] Joly N, Burrows PC, Engl C, Jovanovic G, Buck M (2009). A lower-order oligomer form of phage shock protein A (PspA) stably associates with the Hexameric AAA(+) transcription activator protein pspF for negative regulation. J Mol Biol.

[b19] Lee S-Y, De La Torre A, Yan D, Kustu S, Nixon BT, Wemmer DE (2003). Regulation of the transcriptional activator NtrC1: structural studies of the regulatory and AAA+ ATPase domains. Genes Dev.

[b20] Norby JG (1988). Coupled assay of Na+,K+-ATPase activity. Methods Enzymol.

[b21] North AK, Weiss DS, Suzuki H, Flashner Y, Kustu S (1996). Repressor forms of the enhancer-binding protein NtrC – some fail in coupling ATP hydrolysis to open complex-formation by sigma 54- holoenzyme. J Mol Biol.

[b22] Rappas M, Schumacher J, Niwa H, Buck M, Zhang X (2006). Structural basis of the nucleotide driven conformational changes in the AAA+ domain of transcription activator PspF. J Mol Biol.

[b23] Rappas M, Bose D, Zhang X (2007). Bacterial enhancer-binding proteins: unlocking sigma54-dependent gene transcription. Curr Opin Struct Biol.

[b24] Schumacher J, Zhang X, Jones S, Bordes P, Buck M (2004). ATP-dependent transcriptional activation by bacterial PspF AAA+protein. J Mol Biol.

[b25] Schumacher J, Joly N, Claeys-Bouuaert IL, Aziz SA, Rappas M, Zhang X, Buck M (2008). Mechanism of homotropic control to co-ordinate hydrolysis in a hexameric AAA+ ring ATPase. J Mol Biol.

[b26] Studholme DJ, Dixon R (2003). Domain architectures of sigma54-dependent transcriptional activators. J Bacteriol.

[b27] Tucker NP, D'Autreaux B, Studholme DJ, Spiro S, Dixon R (2004). DNA binding activity of the *Escherichia coli* nitric oxide sensor NorR suggests a conserved target sequence in diverse proteobacteria. J Bacteriol.

[b28] Tucker NP, D'Autreaux B, Yousafzai FK, Fairhurst SA, Spiro S, Dixon R (2008). Analysis of the nitric oxide-sensing non-heme iron center in the norr regulatory protein. J Biol Chem.

[b29] Tucker NP, Ghosh T, Bush M, Zhang X, Dixon R (2010). Essential roles of three enhancer sites in σ^54^-dependent transcription by the nitric oxide sensing regulatory protein NorR. Nucleic Acids Res.

[b30] Wedel A, Kustu S (1995). The bacterial enhancer-binding protein NTRC is a molecular machine: ATP hydrolysis is coupled to transcriptional activation. Genes Dev.

[b31] Whitehall S, Austin S, Dixon R (1992). DNA supercoiling response of the sigma 54-dependent *Klebsiella pneumoniae nifL* promoter *in vitro*. J Mol Biol.

[b32] Yan D, Kustu S (1999). ‘Switch I’ mutant forms of the bacterial enhancer-binding protein NtrC that perturb the response to DNA. Proc Natl Acad Sci USA.

[b33] Zhang X, Wigley DB (2008). The ‘glutamate switch’ provides a link between ATPase activity and ligand binding in AAA+ proteins. Nat Struct Mol Biol.

[b34] Zhang X, Chaney M, Wigneshweraraj SR, Schumacher J, Bordes P, Cannon W, Buck M (2002). Mechanochemical ATPases and transcriptional activation. Mol Microbiol.

[b35] Zhang N, Joly N, Burrows PC, Jovanovic M, Wigneshweraraj SR, Buck M (2009). The role of the conserved phenylalanine in the σ^54^-interacting GAFTGA motif of bacterial enhancer binding proteins. Nucleic Acids Res.

