# Supplementary material for: Nitric oxide-responsive interdomain regulation targets the σ54-interaction surface in the enhancer binding protein NorR
Source: Mol Microbiol. 2010 Sep;77(5):1278–88. doi: 10.1111/j.1365-2958.2010.07290.x (PMC2941729; doi:10.1111/j.1365-2958.2010.07290.x)
Supplement: Supplementary file 1 [file mmi0077-1278-SD1.pdf]

Supporting Information for:

**Nitric oxide-responsive inter-domain regulation targets the $\sigma^{54}$ - interaction surface in the enhancer binding protein NorR**

By **Matthew Bush, Tamaswati Ghosh, Nicholas Tucker, Xiaodong Zhang and Ray Dixon**

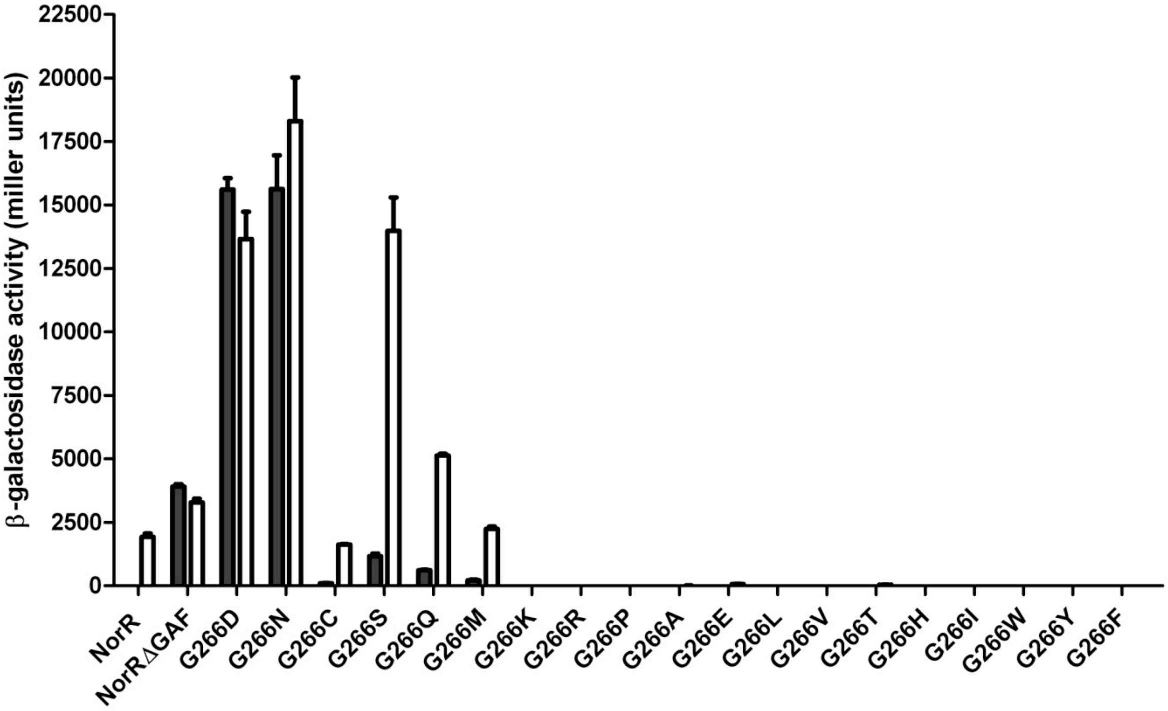


**Figure S1.** Activities of G266 variants *in vivo* as determined by the *norV-lacZ* reporter assay "NorR" represents the wild-type protein and "NorRΔGAF", the N-terminally truncated (Δ1-170) protein. Cultures were grown either in the absence (black bars) or presence (white bars) of 4 mM potassium nitrite, which induces endogenous NO production. Error-bars show the standard error of the three replicates carried out for each condition.

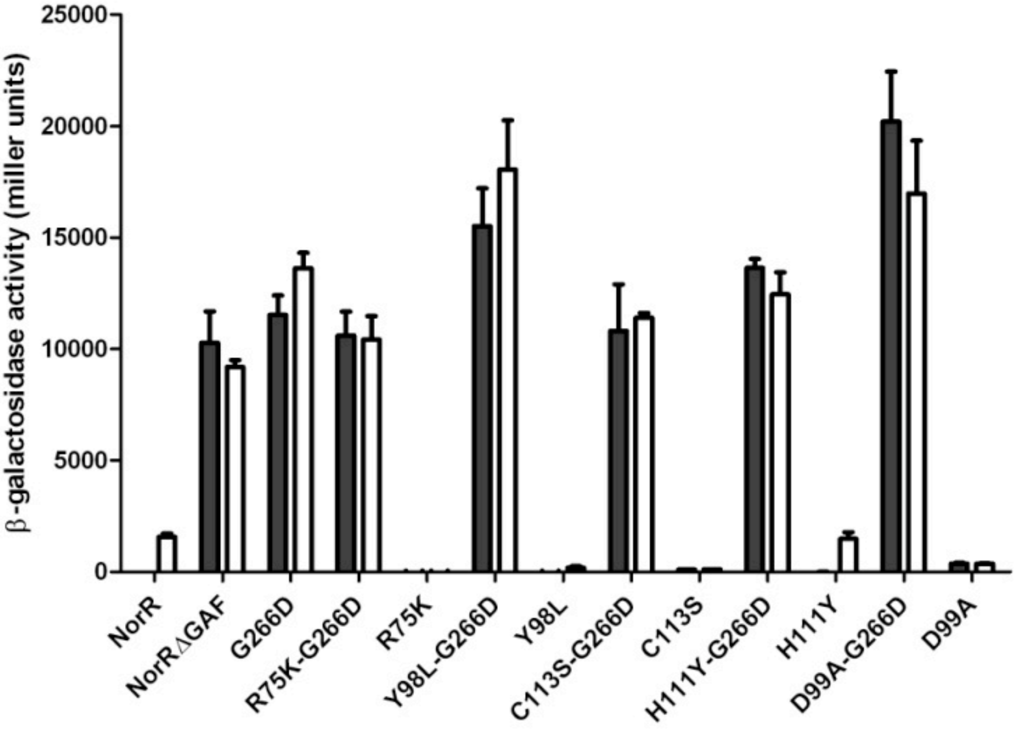


**Figure S2.** Activities of G266 variants *in vivo* when additional amino acid changes are made in the GAF domain to substitute residues that are predicted to coordinate the iron. “NorR” represents the wild-type protein and “NorRΔGAF”, the N-terminally truncated (Δ1-170) protein. Cultures were grown either in the absence (black bars) or presence (white bars) of 4 mM potassium nitrite, which induces endogenous NO production. Error-bars show the standard error of the three replicates carried out for each condition.

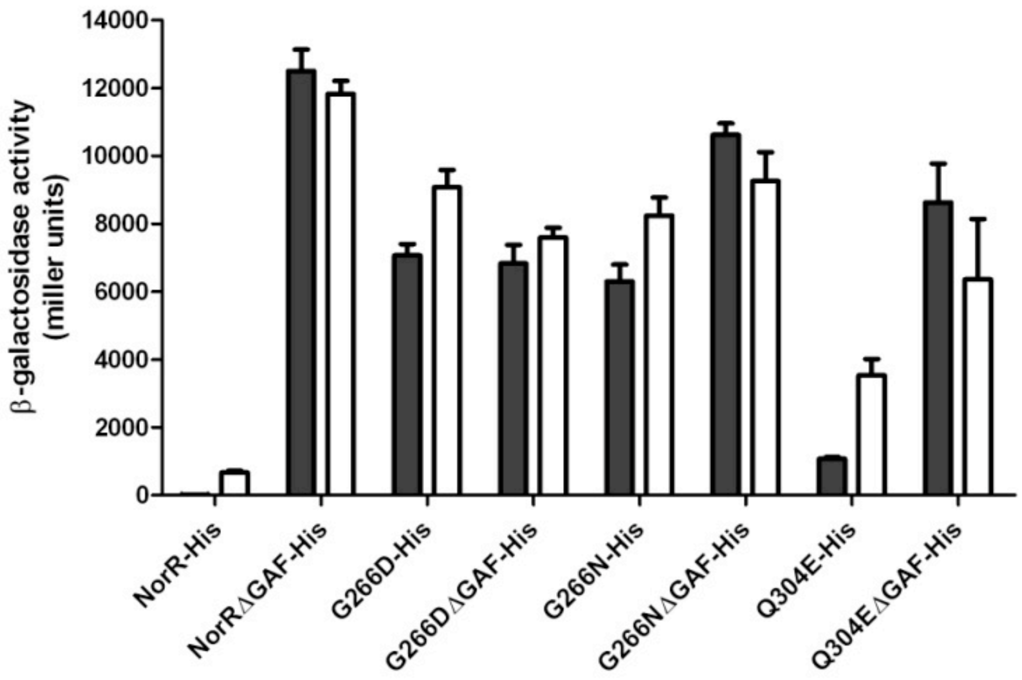


**Figure S3.** *In vivo* activities of the NorR-His, G266D-His, G266N-His and Q304E-His proteins in their full length and N-terminal GAF-truncated forms (Δ1-170) as determined by the *norV-lacZ* reporter assay. Cultures were grown either in the absence (black bars) or presence (white bars) of 4 mM potassium nitrite, which induces endogenous NO production. Error-bars show the standard error of the three replicates carried out for each condition.

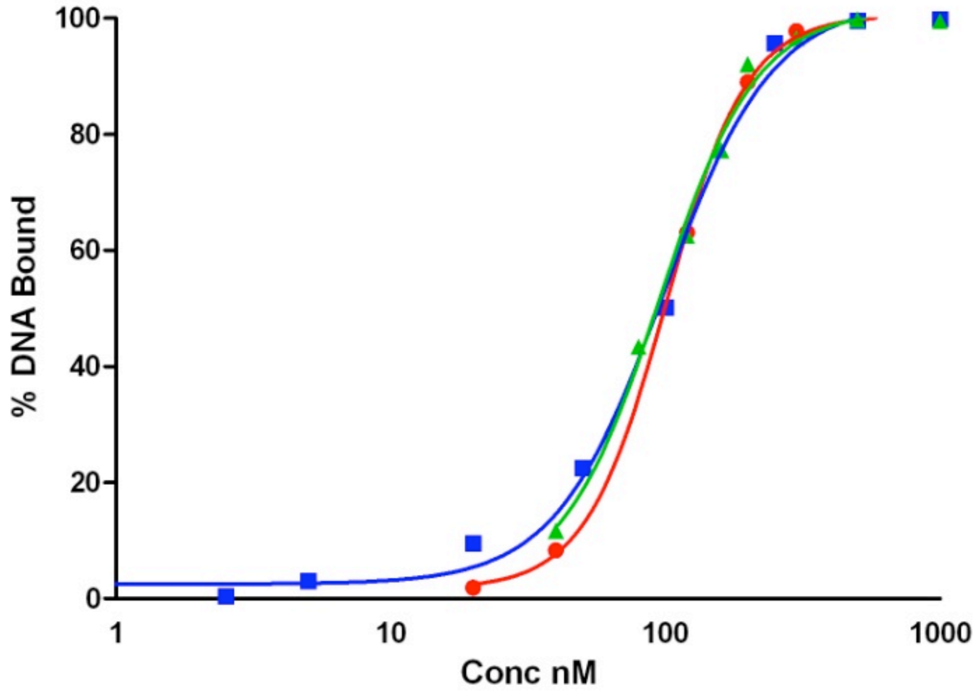


**Figure S4.** Enhancer binding activity of the G266DΔGAF-His (blue squares) and G266NΔGAF-His (green triangles) variants compared to NorRΔGAF-His (red circles) as determined by gel retardation assays. The percentage of fully shifted DNA was quantified using a Fujix BAS 1000 phosphoimager**.** The G266 substitutions do not significantly affect the affinity of NorR for the 361bp fragment of the *norR-norVW* intergenic region that contains the 3 NorR binding sites.

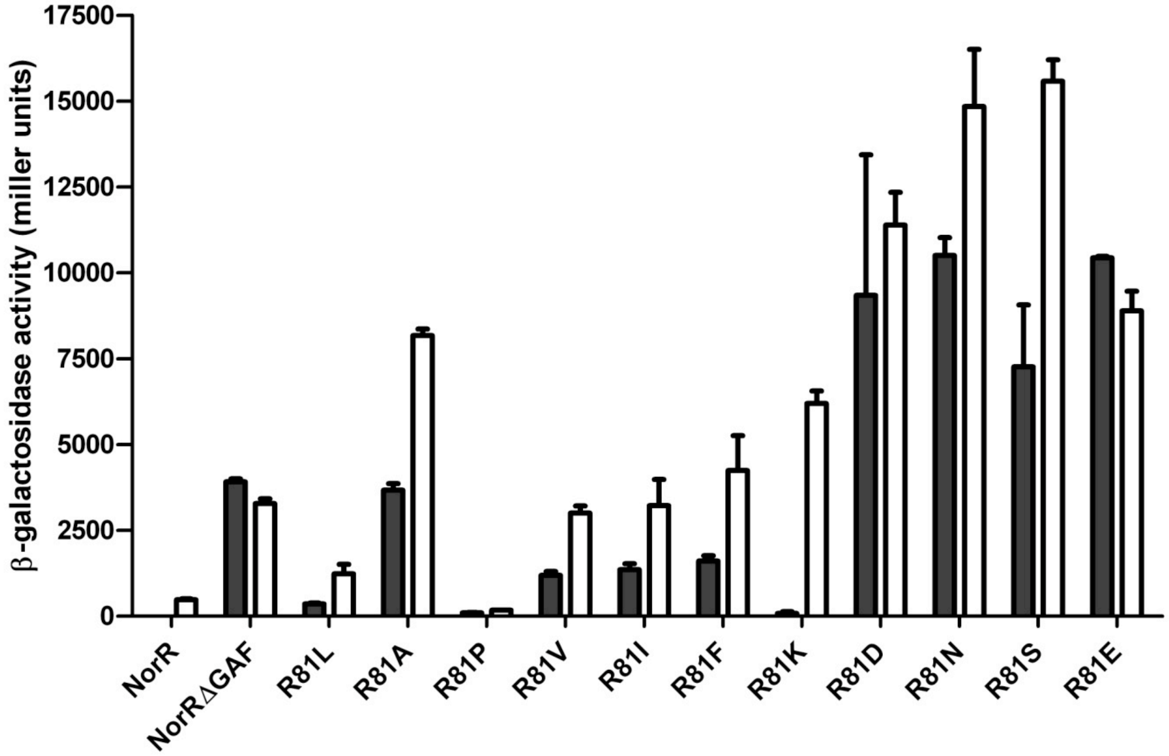


**Figure S5.** Activities of R81 variants *in vivo* as determined by the *norV-lacZ* reporter assay "NorR" represents the wild-type protein and "NorRΔGAF", the N-terminally truncated (Δ1-170) protein. Cultures were grown either in the absence (black bars) or presence (white bars) of 4 mM potassium nitrite, which induces endogenous NO production. Error-bars show the standard error of the three replicates carried out for each condition.

**A**

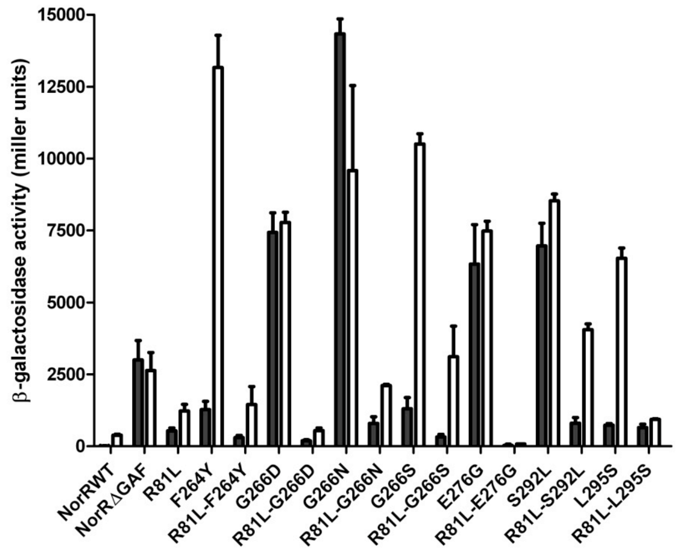


**B**

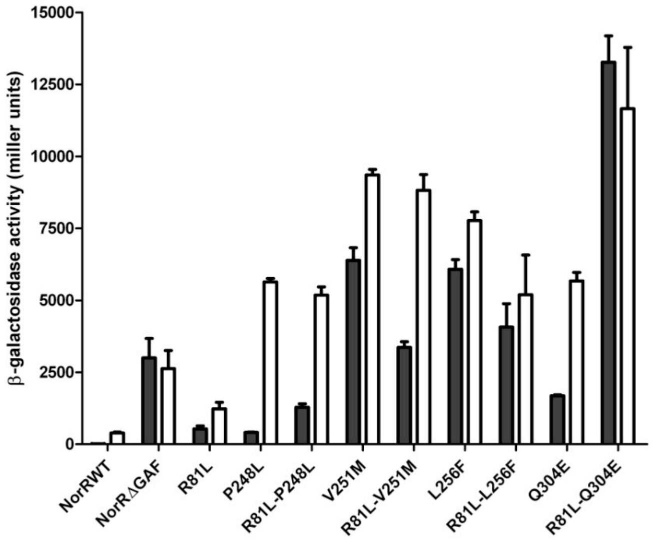


**Figure S6.** Influence of the R81 substitution on the activity of AAA+ domain variants **(A)** NorR AAA+ variants that are effectively suppressed by the R81L substitution in the GAF domain. **(B)** NorR AAA+ variants that are not significantly suppressed by the R81L substitution in the GAF domain. "NorR" is the wild-type protein. "NorRΔGAF" is the N-terminally truncated (Δ1-170) protein. Activities *in vivo* (as determined by the *norV-lacZ* reporter assay) were measured either in the absence (black bars) or presence (white bars) of 4 mM potassium nitrite, which induces endogenous NO production. Error-bars show the standard error of the three replicates carried out for each condition.

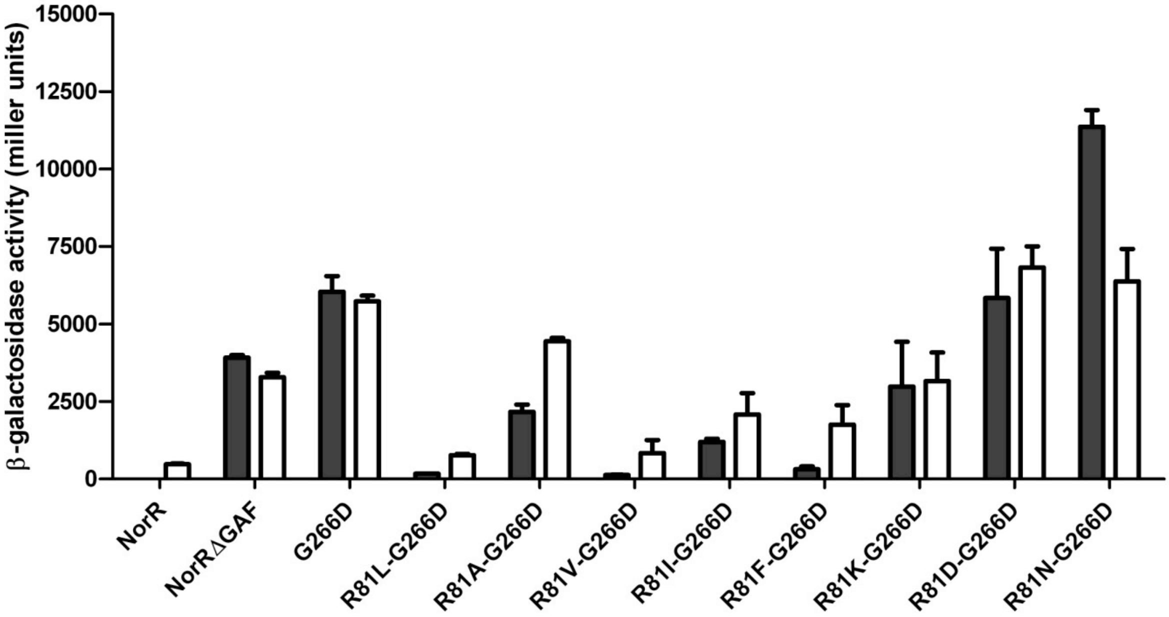


**Figure S7.** Suppression of the constitutive phenotype of the NorRG266D variant by hydrophobic substitutions made at the R81 residue in the GAF domain. "NorR" is the wild-type protein. "NorRΔGAF" is the N-terminally truncated (Δ1-170) protein. Activities *in vivo* (as determined by the *norV-lacZ* reporter assay) were measured either in the absence (black bars) or presence (white bars) of 4 mM potassium nitrite, which induces endogenous NO production. Error-bars show the standard error of the three replicates carried out for each condition.

A

B

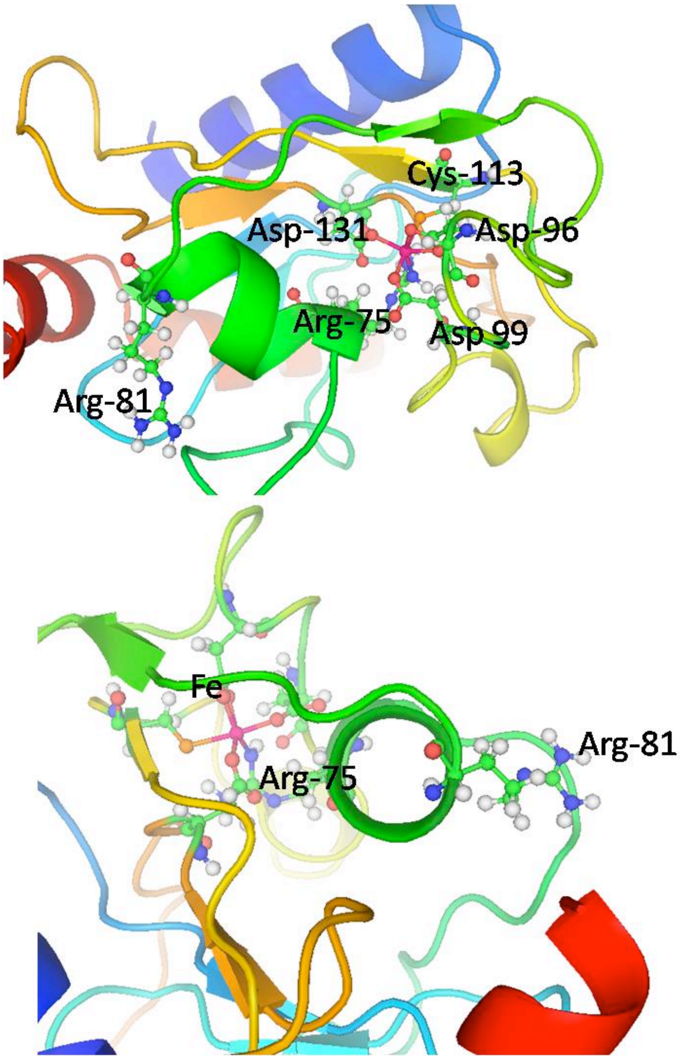


**Figure S8.** The R81-mediated mechanism of inter-domain repression (**A)** Structural model of the GAF domain of NorR based on the GAF-B domain of 3',5'-cyclic nucleotide phosphodiesterase (Tucker et al., *J Biol Chem* 283:908-918, 2008) showing the iron centre and proposed ligands, relative to the R81 residue. (**B**) The model predicts that the residue is surface exposed and may therefore be well positioned for AAA+ contact.
